# Case of Spasmodic Constriction of the Œsophagus

**Published:** 1852-08

**Authors:** B. Palais


*Case of Spasmodic Constriction of the Œsophagus.* By Dr. B. Palais.—The patient was a young man, aged 21, who had intermittent fever. Dr. Palais was called to him in the hospital at Montmorail, on December 22, 1851, where he found him in the following condition:

The skin was in general cold; there was trembling of the whole body; the pulse was 60; the jaws were contracted; deglutition was impossible, and liquids produced severe pain in the pharynx, and caused painful efforts to swallow, which congested the face; there was difficulty in protruding the tongue; the front of the neck was tender, and the movements of this part were impaired, while the limbs and back could be bent; the urine was scanty; the patient tossed himself about in bed; he understood questions, but could only answer them imperfectly. Sinapisms were applied several times to the lower limbs; warmth was applied to the body; and ether mixture was given; and the patient was bled from the arm.

*December* 23. The same state continuing, the bleeding was repeated.

*December* 25. The patient was calm; he could swallow a little, but with difficulty, so that it seemed as if the fluids were passing through a filter. The pulse was 72; the skin was regaining its normal heat. He complained of pain at the upper part of the sternum, and also between the lower ribs on the left side. Nothing abnormal was detected in the thorax by auscultation.

*December* 28. The dysphagia had gradually ceased, and there now appeared slight attacks of ague, which continued till the 31st, and then yielded to sulphate of quinine.—*Ibid.*

